# Simulation-based mastery learning compared to standard education for discussing diagnostic uncertainty with patients in the emergency department: a randomized controlled trial

**DOI:** 10.1186/s12909-020-1926-y

**Published:** 2020-02-19

**Authors:** Danielle M. McCarthy, Rhea E. Powell, Kenzie A. Cameron, David H. Salzman, Dimitrios Papanagnou, Amanda MB. Doty, Benjamin E. Leiby, Katherine Piserchia, Matthew R. Klein, Xiao C. Zhang, William C. McGaghie, Kristin L. Rising

**Affiliations:** 1 Department of Emergency Medicine, Northwestern University Feinberg School of Medicine, 211 East Ontario, Suite 200, Chicago, IL 60611 USA; 2 Division of General Internal Medicine and Geriatrics, Northwestern University, Philadelphia, PA USA; 3 Division of General Internal Medicine, Department of Medicine, Northwestern University Feinberg School of Medicine, Chicago, IL USA; 4 Department of Medical Education, Northwestern University Feinberg School of Medicine, Chicago, IL USA; 5 Department of Emergency Medicine, Thomas Jefferson University, Philadelphia, PA USA; 6 Division of Biostatistics, Department of Pharmacology and Experimental Therapeutics, Thomas Jefferson University, Philadelphia, PA USA

**Keywords:** Uncertainty, Medical education, Simulation based mastery learning, Emergency medicine, Communication, Emergency department

## Abstract

**Background:**

Diagnostic uncertainty occurs frequently in emergency medical care, with more than one-third of patients leaving the emergency department (ED) without a clear diagnosis. Despite this frequency, ED providers are not adequately trained on how to discuss diagnostic uncertainty with these patients, who often leave the ED confused and concerned. To address this training need, we developed the Uncertainty Communication Education Module (UCEM) to teach physicians how to discuss diagnostic uncertainty. The purpose of the study is to evaluate the effectiveness of the UCEM in improving physician communications.

**Methods:**

The trial is a multicenter, two-arm randomized controlled trial designed to teach communication skills using simulation-based mastery learning (SBML). Resident emergency physicians from two training programs will be randomly assigned to immediate or delayed receipt of the two-part UCEM intervention after completing a baseline standardized patient encounter. The two UCEM components are: 1) a web-based interactive module, and 2) a smart-phone-based game. Both formats teach and reinforce communication skills for patient cases involving diagnostic uncertainty. Following baseline testing, participants in the immediate intervention arm will complete a remote deliberate practice session via a video platform and subsequently return for a second study visit to assess if they have achieved mastery. Participants in the delayed intervention arm will receive access to UCEM and remote deliberate practice after the second study visit. The primary outcome of interest is the proportion of residents in the immediate intervention arm who achieve mastery at the second study visit.

**Discussion:**

Patients’ understanding of the care they received has implications for care quality, safety, and patient satisfaction, especially when they are discharged without a definitive diagnosis. Developing a patient-centered diagnostic uncertainty communication strategy will improve safety of acute care discharges. Although use of SBML is a resource intensive educational approach, this trial has been deliberately designed to have a low-resource, scalable intervention that would allow for widespread dissemination and uptake.

**Trial registration:**

The trial was registered at clinicaltrials.gov (NCT04021771). Registration date: July 16, 2019.

## Background

Diagnostic uncertainty is a frequent occurrence in the emergency department (ED), with at least 37% of patients treated in the ED discharged without a pathologic diagnosis [1]. For example, a patient may present to the ED with a complaint of chest pain. After potentially dangerous diagnoses are excluded, the patient may then be discharged with a discharge diagnosis of “chest pain,” a symptom-based diagnosis. In the absence of a specific cause of her symptoms, she is tasked with navigating future care in the setting of diagnostic uncertainty. Faced with this uncertainty, patients often experience fear, with negative impacts on mental and physical health in the post-discharge period [2–4]. Although diagnostic uncertainty is frequent in emergency care settings, medical professionals have minimal guidance on how to communicate effectively to help patients transition home safely with such uncertainty. Among emergency medicine trainees, 99% reported encountering challenges discharging patients with diagnostic uncertainty at least sometimes, and 43% have encountered this challenge ‘often’ or all of the time [5].

Transitions of care are well established as a high-risk period for patient safety events [6–13]. Effective patient communication is essential in promoting safety during care transitions [14–16], yet studies of verbal ED discharge have found that instructions are often incomplete, with few opportunities for patients to ask questions or confirm understanding [17]. Numerous research efforts target reducing harm at care transitions [6–11, 18], including efforts to improve discharge coordination [19] and communication of discharge instructions [20]. Communication of discharge diagnosis, prognosis, treatment plan, and expected course of illness is important to high quality ED discharge [21], and prior work on communication during care transitions has focused on improving the content, delivery, and comprehension of discharge instructions [15, 22]. These efforts have included provision of diagnosis-specific discharge instructions [23, 24]. However, for patients discharged from the ED with diagnostic uncertainty, no standardized instructions and no standard approach to discuss uncertainty currently exist.

We developed an educational curriculum using simulation-based mastery learning (SBML) for resident physicians to guide patient-provider communication in the setting of uncertainty at discharge. SBML is a competency-based educational approach that allows learners to develop skills through deliberate practice, resulting in very high levels of performance outcomes, with little variation in outcomes among learners [25, 26]. In SBML, learners are required to meet or exceed a predetermined minimum passing standard (MPS) on a skills-based checklist or knowledge examination before completion of training. There is a large and growing body of evidence supporting the use of SBML as an educational modality for acquiring technical and procedural skills [27–32]. However, SBML has less frequently been used for acquisition of communication and interpersonal skills [33, 34]. SBML is time and resource intensive, as one of the main means by which trainees acquire mastery is through repeated deliberate practice. A SBML curriculum relevant to a communication task requires the additional specialized resource of 1-on-1 time with a standardized patient. Such increased time with standardized patients raises educational costs and increases the need for coordination with trainees’ clinical schedules. To address these intensive resource needs, Issenberg et al. suggested that perhaps skill acquisition and retention “can be achieved with different, often less costly and more flexible, simulation modalities such as virtual patients” [35].

Research groups have begun to develop educational designs that use video-based observational platforms [36, 37] and have found that these designs improve efficiency of skill acquisition within SBML [38]. In parallel, there is a growing body of research on the role of virtual patients (defined as “an interactive computer simulation of real-life clinical scenarios for the purpose of healthcare and medical training, education, or assessment”) [39]. A less frequently explored modality is a hybrid approach to simulation using standardized patients remotely. To our knowledge, only four studies have evaluated the use of remote standardized patients [40–43]; however, this method has promise as it minimizes the local resources needed, builds upon known strengths of standardized patients and retains the fidelity of a human interaction, yet does not require the intensive computer programming of developing virtual patients.

This randomized controlled waitlist trial will evaluate the acquisition and retention of communication skills in discussing diagnostic uncertainty at the time of ED discharge among participants exposed to remote standardized patient practice and two web-based educational tools (a curriculum and a game), compared to usual education. To our knowledge, this is the first multi-center randomized trial of SBML designed to teach communication skills using remote standardized patients to complete the deliberate practice.

## Methods/design

The study will use a randomized controlled waitlist trial design with two study arms. The intervention arm exposes emergency medicine (EM) resident physicians to a two-part intervention, entitled the Uncertainty Communication Education Module (UCEM), while residents in the control arm receive delayed exposure to UCEM. The study will test the effectiveness of UCEM and remote deliberate practice in teaching residents to achieve mastery of uncertainty communication, as assessed by the Uncertainty Communication Checklist (UCC) [44]. UCEM has two components: 1) a web-based interactive module; and 2) a smart-phone-based game. Both components teach and reinforce communication skills that can be applied clinically when treating patients who are discharged from the ED with diagnostic uncertainty.

The UCEM is coupled with deliberate practice sessions with remote standardized patients. Participants will be assessed using the Uncertainty Communication Checklist (UCC) [44], a tool to guide the assessment of trainees when discharging patients from the ED in the setting of diagnostic uncertainty. Both the UCEM and the UCC were developed through an iterative process of feedback with experts and patients with the goal of educating providers about diagnostic uncertainty in the acute care setting, and improving their skills in discussing this topic with patients.

All study procedures were reviewed and approved by the Thomas Jefferson University Internal Review Board (IRB), with an IRB authorization agreement between Northwestern University and Thomas Jefferson. The trial was registered at clinicaltrials.gov (NCT04021771). The authors hypothesize that resident physicians who receive the UCEM intervention and complete deliberate practice will be significantly more likely to achieve mastery at the initial post-test in this communication skill compared to residents in the control arm.

### Study design and setting

Rather than employing a pre- / post-test design, as is often used in SBML, we will use a randomized controlled waitlist study design. The waitlist design allows us to maintain the goal of a mastery-learning curriculum, eventually affording all resident learners the access to the intervention and the opportunity to achieve mastery, but simultaneously allows for rigorous scientific evaluation.

The study will take place at two EM residency-training programs in the United States (i.e., Philadelphia and Chicago) during the 2019–2020 academic year (study ongoing). All emergency medicine residents, post-graduate years (PGY) 1 through 4 (i.e., PGY 1 through 3 in Philadelphia, and PGY 1 through 4 in Chicago) are eligible for participation. There are no exclusion criteria. The educational activities are being integrated into the formal residency curriculum at each site; therefore, residents are being consented for use of their data, but if they decline consent, they will nonetheless complete the simulation testing and educational components of the curriculum. A study team member will obtain written consent from all study participants for use of their data. Residents at each site will be randomly assigned to a study arm using a computer-generated randomization schema stratified by stage of training (i.e., junior resident [PGY 1–2], senior resident [PGY 3–4]) and training site (i.e., Philadelphia, Chicago).

### Study visits

**Fig. 1:**
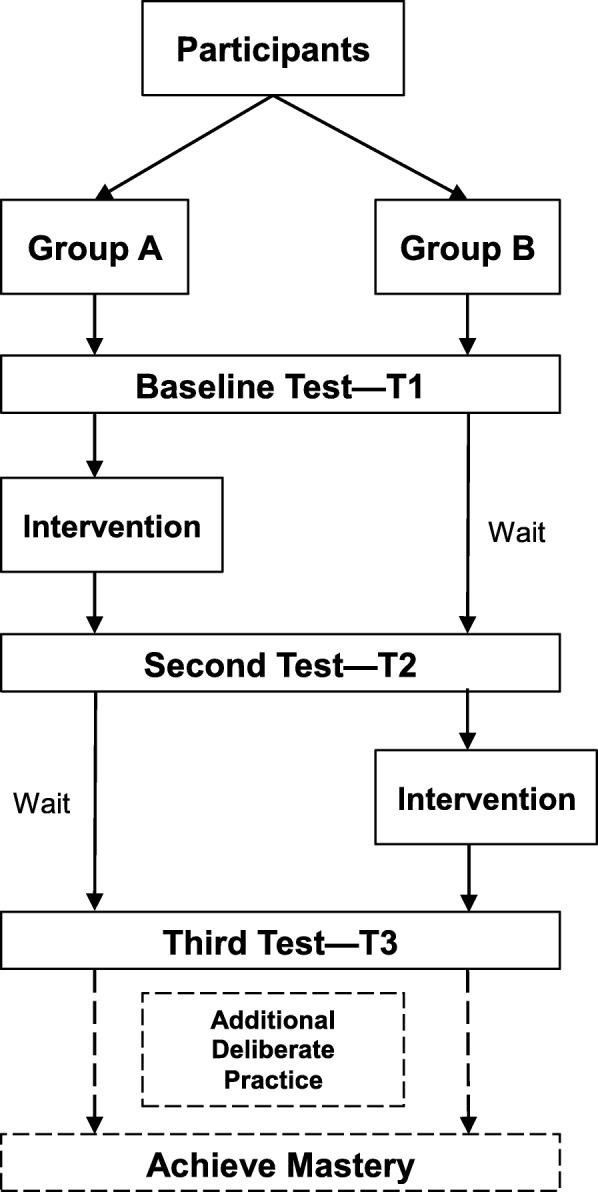
Participants flow through study

Three study test visits are planned for all participants at the following time points: T1 (baseline), T2 (8–12 weeks post-baseline), and T3 (16–24 weeks post-baseline) (Fig. 1). The T1 visit will consist of the baseline test for all participants, during which they will complete a simulated encounter with an in-person standardized patient to establish a baseline score on the UCC. Participants who are randomized to the Group A (immediate intervention) will receive immediate and detailed feedback on their performance in the encounter, which will include an introduction to the UCC. Following the baseline testing, each participant will receive a group allocation. The allocation sequence will be generated in advance by the study statistician and implemented via sequentially numbered, sealed, opaque envelopes which will be opened by research personnel while the participant is completing their baseline simulated encounter. Participants in Group A will be given access to UCEM (the intervention) and will be scheduled to participate in the telehealth remote deliberate practice sessions with a standardized patient. Participants in Group B, allocated to delayed access to UCEM, will not receive any study-related intervention prior to T2.

**Table 1:** Table caption

	Study Period					
	Enrolment	Post-allocation				Closeout
TIMELINE	M0-M1	M2-3	M4-5	M6-7	M8-9	M10
TIMEPOINT	T1		T2		T3	
ENROLMENT:						
Eligibility screen	X					
Informed consent	X					
Allocation	X					
Demographic data	X					
INTERVENTIONS:						
UCEM Access (Group A)		X				
Deliberate Practice (Group A)		X				
UCEM Access (Group B)				X		
Deliberate Practice (Group B)				X		
Additional Practice for those not achieving mastery						X
ASSESSMENT:						
*Performance* on Simulated Encounter (UCC score)^a^	X		X		X	
Surveys on *experience* with simulated encounter, UCEM & deliberate practice (Group A)			X			
Surveys on *experience* with simulated encounter, UCEM & deliberate practice (Group B)					X	
Surveys on *knowledge transfer* into clinical practice					X	

*Abbreviations*: *M* Month, *UCEM* Uncertainty Communication Education Module, ^a^primary outcome, *UCC* Uncertainty Communication Checklist

The T2 visit will be scheduled approximately 8–12 weeks after T1, and will consist of another in-person test encounter with a standardized patient for both Group A and B. The waitlist approach allows for evaluation of the impact of exposure to the baseline test, UCEM and deliberate practice in Group A versus baseline testing alone in Group B. Although T1 is a baseline “test”, often times a test can act as an intervention by stimulating subsequent reflection and self-directed practice of a skill during routine clinical activities.

Following the T2 visit, participants in Group B will be given access to UCEM and scheduled for telehealth remote deliberate practice sessions with standardized patients. Both groups will return for a third study test visit (T3) approximately 8–12 weeks after T2. All study test visits will involve participants completing a video-recorded simulated encounter with in-person standardized patients, although the clinical scenarios will vary each visit to maximize participants’ exposure to different scenarios. Participants will be assigned a score on the UCC for their performance for each visit, with the primary endpoint being achieving the MPS (binary, yes/no) [45]. Any participant not achieving mastery upon the completion of T3 will be given additional opportunities to practice and retest until mastery is achieved.

Participants will complete surveys at each of the study test visits (T1-T3) to collect demographic information and/or feedback on their experiences with the simulation encounters and educational curriculum. Participating residents will be compensated $75 for sharing their data and their time with completing surveys ($25 at initial enrollment and $50 upon completion of all study activities). Trained research assistants will enter all survey data and UCC scores into an electronic REDCap (Research Electronic Data Capture) database under study identification numbers which will be used to maintain confidentiality [46] (Table 1).

### Simulation cases

Simulation cases intentionally represent common symptom-based diagnoses. A total of sixteen cases are included (i.e., four reserved for testing, eight for deliberate practice, four held in reserve if needed). Each case includes a case history, a normal physical examination, and normal laboratory results with variable degrees of testing performed in the ED (e.g., abdominal pain with blood and urine tests completed versus abdominal pain with computed tomography (CT) completed). Participating residents will be provided with the instructions to: 1) “update the patient” on his/her results; and 2) “discharge the patient” from the ED. They will further be informed that the simulation will be focused on communication and not medical decision making, clarifying that additional testing or admission to the hospital should not be performed.

Standardized patients will complete a training session to learn about the checklist and case content, using multiple modalities including video recordings paired with group discussions and role-plays. In addition to cases representing a range of symptoms, the standardized patients will be assigned an “emotional state” for each case (i.e., reassured, confused, anxious/nervous, or inquisitive). Cases were written by emergency physician experts in simulation education, and revised by team members with both clinical and communication expertise. All cases were designed to be of equal difficulty. Cases were pilot tested and further refined prior to the start of the study.

### UCEM intervention

The UCEM intervention includes an online educational module with links to reading and an interactive smartphone-based game. Participants will receive the intervention at one of two time periods during the study depending on their group assignment (between T1 and T2 for Group A, between T2 and T3 for Group B). During the intervention period, participants may complete the online educational module and smartphone-based game independently at a pace and frequency of their choosing. They may return to the module and game as frequently as they wish.

**Fig. 2:**
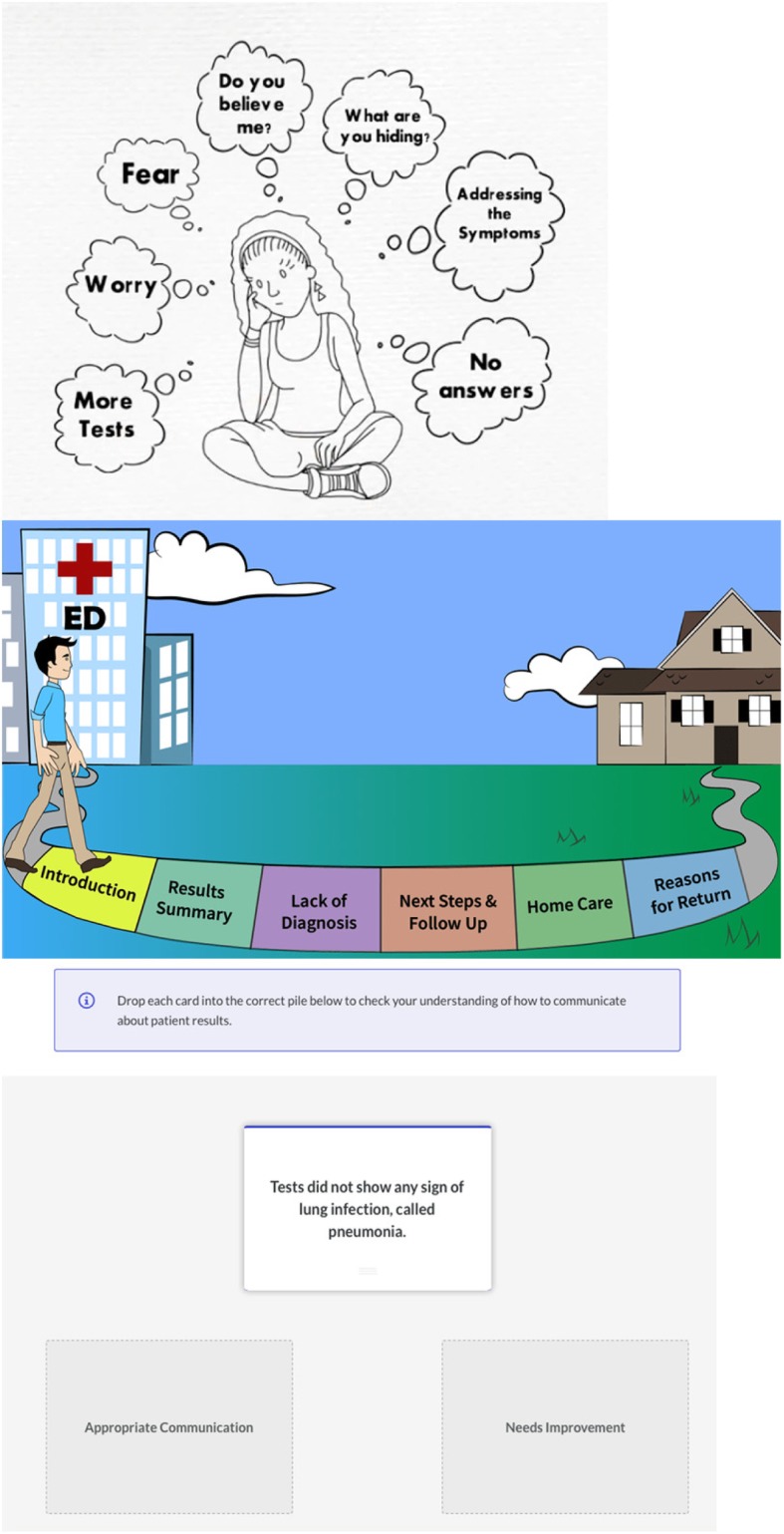
Screenshots from online educational module of UCEM

The online educational module introduces the concept of diagnostic uncertainty, explains the UCC, and includes a variety of tools to support knowledge retention including matching games, video vignettes, and sample discharge conversation content presented via audio-clips. The online educational module was developed by the study team in collaboration with the Center for Teaching and Learning at Thomas Jefferson University. The online educational module is available at https://rise.articulate.com/share/7HtSck8Gw2zbg56yeO356oV0VrN17LWZ#/ (Password: Uncertainty); screenshots from the curriculum are also shown in Fig. 2.

**Fig. 3:**
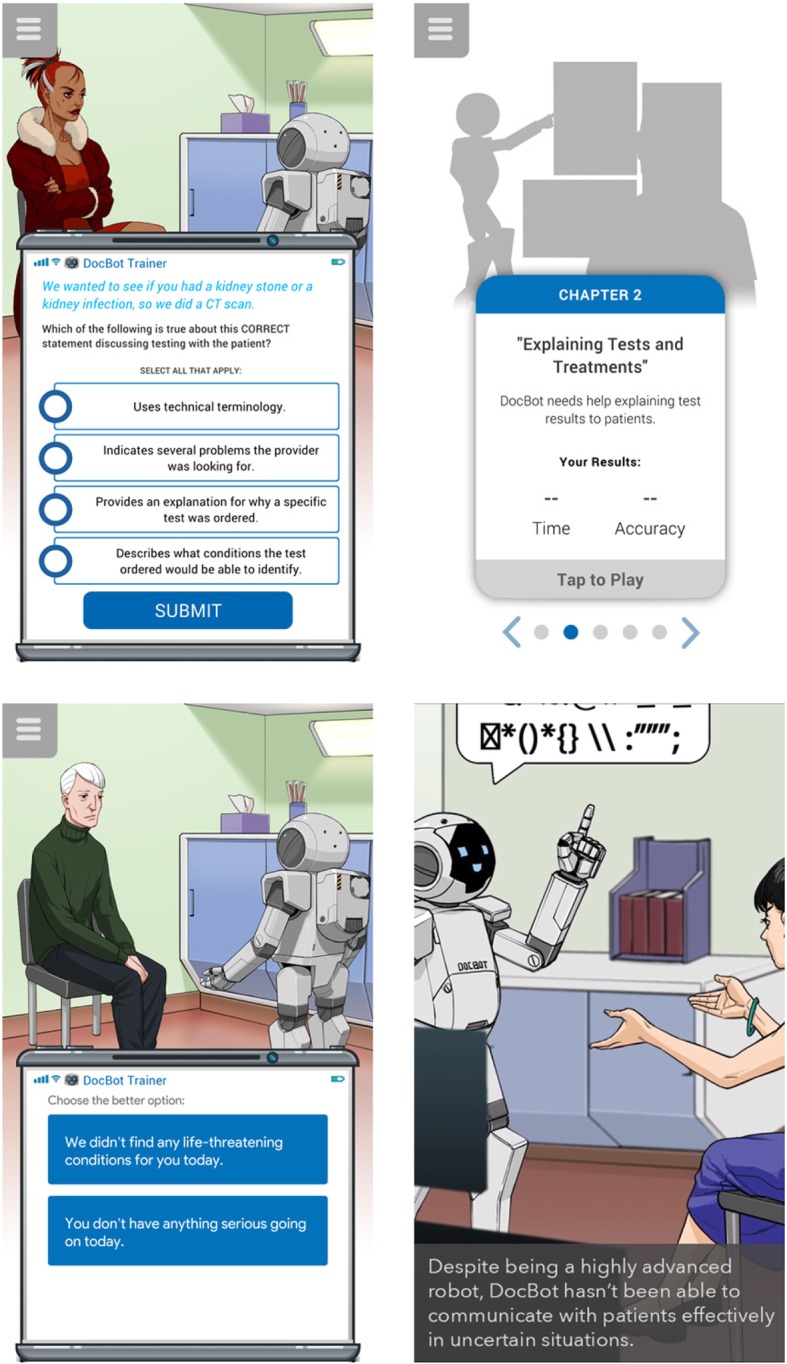
Screenshots from interactive smart-phone based game of UCEM

The interactive game provides learners with a low-stakes environment to reflect on and practice their word choice during discharge conversations. The game includes a series of discharge scenarios. For each scenario, the game displays two choices of phrases relevant to the discharge conversation and asks the learner to select the “better” phrase. Follow-up questions ask the learner to select reasons why a specific phrase is the less/more optimal choice (e.g., “it did not use lay language” or “provides an explanation for why a specific test was ordered”) to reinforce the items of the UCC. Learners are awarded points for their performance. Scores are displayed anonymously on a “leaderboard.” As with the online educational module, participants will be able to play and return to the game as frequently as they desire. Jump Simulation-OSF HealthCare developed the interactive game with input from the study team. The interactive game can be downloaded free from the mobile application stores (i.e., Apple Store) on both Apple and Android™ platforms via the following links: (http://bit.ly/ucomm-apple or http://bit.ly/ucomm-android). Screenshots from the game are show in Fig. 3.

### Telehealth remote deliberate practice

After accessing the UCEM, participants who did not meet the MPS at baseline will be required to schedule at least one telehealth-mediated remote deliberate practice session. Unlike traditional in-person simulation encounters, participants will not be required to report to a dedicated simulation center; rather, residents may participate in the encounter from a location of their own choosing. These sessions will be completed using the Zoom platform [(2019) Zoom Cloud Meetings: A video-conferencing tool (Version 4.4.6). Available from https://zoom.us]. After each practice session, participants will receive immediate and detailed feedback from the standardized patient regarding their performance. Participants will have the option to complete additional deliberate practice sessions before their subsequent test visit (either T2 or T3) during which they will be officially scored on the UCC. After each remote deliberate practice session, participants will be asked whether they would like to complete additional remote deliberate practice sessions. Case scenarios used for deliberate practice will be distinct from case scenarios used for testing (i.e., different medical complaints), although of similar case complexity.

### Outcomes and measures

The analysis of the trial will report outcomes in three different domains, derived from Kirkpatrick’s *Four Level Evaluation Model* [47].

#### Reaction: participants’ experiences

For this first domain, we will assess participants’ reaction to the educational curriculum and intervention. Although this first domain does not measure the impact of the intervention, it is nonetheless important because holding a learner’s interest and attention is necessary for the success of the teaching intervention. All participants will complete surveys to gather feedback on the simulation experience, the UCEM intervention, and the remote deliberate practice sessions using a combination of open- and closed-ended questions.

#### Learning: performance on simulation

The second domain will assess learning. To measure the curriculum effectiveness in the learning domain, we will report the data from the two-arm randomized controlled waitlist trial, as detailed above. Our primary outcome of interest will be the achievement of the MPS at T2.

Performance on the simulation encounter will be rated at all study visits using the MPS derived previously for the UCC [45]. The primary outcome of interest for this trial is the participant score on the UCC at T2. The UCC is a 21-item checklist assessing if uncertainty was addressed in each step of the discharge communication process and includes the following major categories: Introduction, Test Results/ED summary, No/Uncertain Diagnosis, Next Steps/Follow up, Home Care, Reasons to Return, and General Communication Skills (Appendix). Trained standardized patients will complete UCC ratings immediately following the simulation sessions. Standardized patients will be blinded to study arm allocation, as well as the MPS, at the time of the simulated encounter and scoring; both participants and standardized patients will be unblinded after scores have been collected in order to provide feedback to those in the immediate intervention group. Additionally, a research team member, blinded to the participant’s study arm, will complete ratings for at least a 50% random sample of the videotaped encounters to demonstrate reliability of scoring [48].

As previously described, SBML results in highly reliable achievement of mastery; however, individual trainees may vary in the amount of practice needed to achieve mastery. In order to assess the differences in participants’ effort required to achieve mastery, we will track the number of testing sessions required by each participant to achieve mastery. Trainees who do not achieve mastery at T2 and T3 will have additional deliberate practice and testing until mastery is achieved. Secondary outcomes of interest in the learning domain include: the number of deliberate practice sessions and the change in pass rate on the UCC from T2 to T3 within groups. This metric will assess retention of mastery in the intervention group and will be a supplemental assessment of the interventions’ efficacy in the delayed intervention (control) group.

Additionally, game access will be tracked; frequency of online module use will be measured via self-reporting. These data will be used to evaluate the relationship between access rates and time to achieve mastery.

#### Transfer: skill utilization in clinical practice

For the third domain, transfer of knowledge, we will contact participants after completion of the education curriculum to inquire about their use of the new communication skills in clinical practice. Topics of questions will include: frequency of encounters with diagnostic uncertainty, comfort level in discussing this topic with patients, patients’ reactions to the conversation and request for anecdotes on patient encounters that could inform future training.

The fourth level of Kirkpatrick’s model, “Results,” will not be assessed in this study due to the timeframe and scope of the funding mechanism.

#### Participant characteristics

All participants will complete a survey after completion of the simulation encounter at T1 with items including basic demographic characteristics (i.e., age, gender, race/ethnicity, post-graduate year). Additionally, participants will be asked open-ended questions, including how often they encounter clinical scenarios with diagnostic uncertainty in their clinical practice, how comfortable they are in having these conversations, what strategies they have used and found successful, and if they have had prior training on this topic.

### Sample size calculation

As this will be the first application of the UCC, it is difficult to estimate a baseline pass rate for power calculations. Prior studies of other clinical skills in EM trainees show pre-test pass rates in the 45–70% range, albeit not in an SBML testing setting [32]. Pre-test communication skills vary widely in clinical practice, with most metrics reporting “excellent” ratings in the 50–80% range. In the ED setting, items related to “allowing patient to ask questions,” which are likely most relevant to the UCC score, are lower, with only 50% of clinicians being rated as excellent [49, 50]. Although participants may “pass” individual items on the UCC at a rate of 60% at baseline, we estimate a baseline performance of 3% of residents meeting the MPS on the pre-test. This estimate was based on prior SBML studies in communication with pass-rates all less than 10% [29, 33]. We expect waitlist subjects to have only a slight improvement in meeting MPS at T2 resulting in a 10% pass rate. Randomizing 110 participants and conservatively estimating a near 90% retention at the Period 2 assessment (*n* = 100, 50 per group), we will have greater than 87% power to detect a difference of 25% between study groups at T2 (intervention pass rate of 35%) assuming a two-sided Type I error of 5%. To optimize participant enrollment and retention, several strategies will be deployed including wide availability of simulation dates and times, email and text reminders for scheduled sessions, and financial incentive as noted above.

### Analysis

**Fig. 4:**
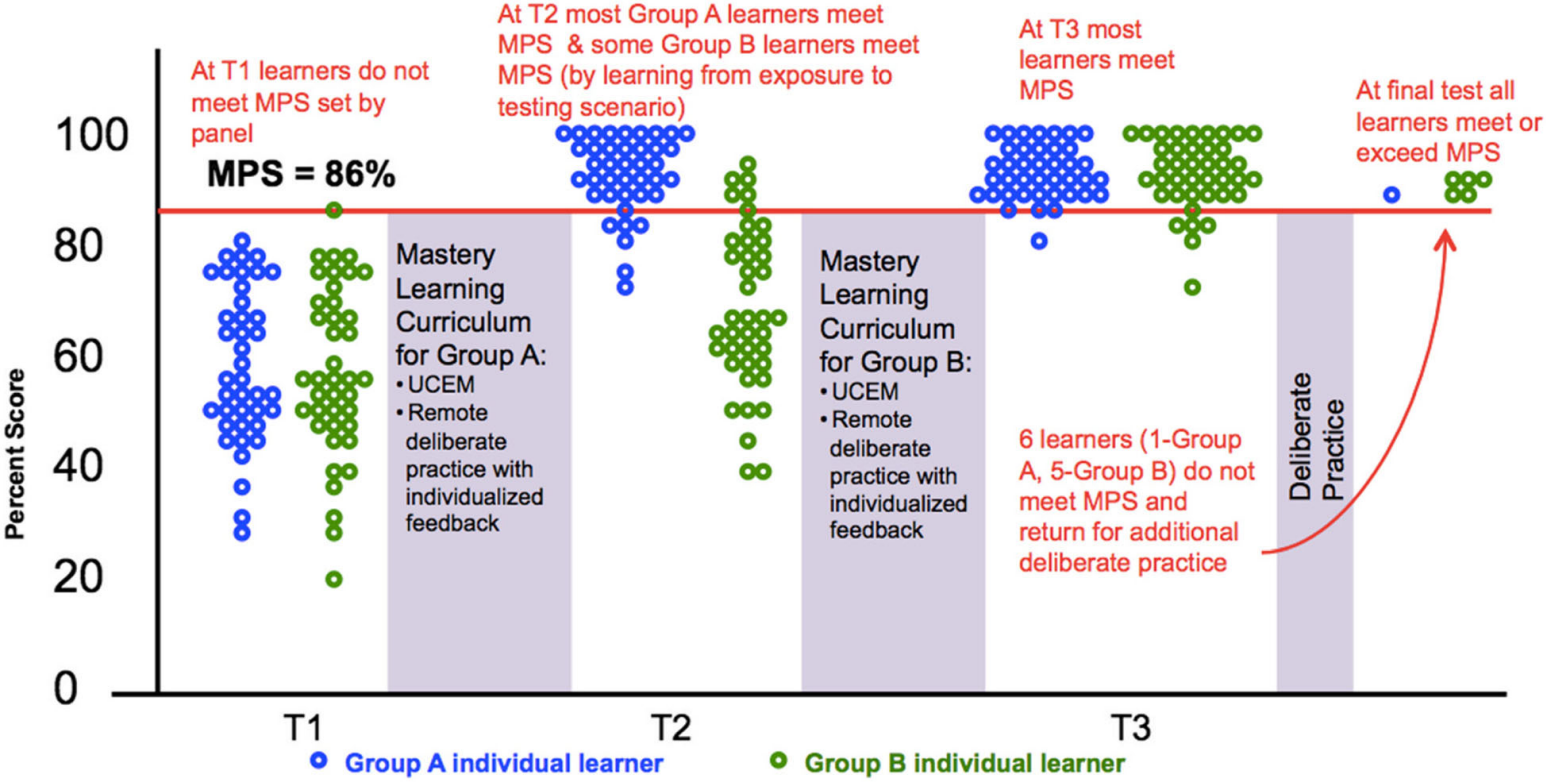
Sample of anticipated results demonstrating individual scores at each testing time point

The primary outcome of interest for the trial is within Kirkpatrick’s learning domain, and is the percentage of residents in each arm who meet or exceed the MPS of the UCC at the T2 assessment. Agreement between standardized patients and the study team rater will be estimated using the Kappa coefficient. We will use a logistic regression analysis to compare groups with respect to the primary outcome at T2. Secondary analysis will consider change within groups from T2 to T3 separately using McNemar’s test. In Group A, this will be a test of whether mastery is retained versus not retained. In Group B, this will be a supplemental test of the intervention’s efficacy. Association between number of remote deliberate practice sessions completed and achieving a passing score will be evaluated using logistic regression, adjusting for the stratification factors of site and stage of training. Among those who pass, the association of the number of remote deliberate practice sessions completed with baseline characteristics will be evaluated using Poisson regression. The outcome of the trial will be reported according to the recently published Reporting Master Education Learning in Medicine (ReMERM) guidelines [51]. Figure 4 illustrates how results of SBML are often reported visually.

Open-ended responses to participants’ experiences “reaction” outcomes and use in current practice “transfer” outcomes will be analyzed qualitatively using content and constant comparative method to evaluate for emergent themes. Closed-ended questions related to their reaction and transfer will be reported with descriptive statistics.

## Discussion

Patients’ understanding of the care they received and their next steps upon leaving an acute care setting have implications on care quality, safety, and patient satisfaction, especially when they are discharged from the ED without a definitive diagnosis. Developing a patient-centered way to communicate is a necessary step to improve safety of acute care discharges in the setting of diagnostic uncertainty. Use of a SBML curriculum focused on diagnostic uncertainty has many potential benefits for improving communication skills; however, SBML is a resource intensive educational approach. Our study attempts to address the high resource of deliberate practice in SBML through the novel use of a telehealth-facilitated approach for practice sessions. We anticipate that this platform will be favorable for our trainees, and may have broader applicability for other communication-related educational curricula.

Findings from this trial may shape residency training of communication skills related to diagnostic uncertainty. A benefit of the UCEM intervention is the potential for widespread dissemination with use of minimal resources, including remote access to deliberate practice via a telehealth video platform and web-based curricular materials. Additionally, while our study focuses on a specific clinical scenario (i.e., discharge from the ED in the setting of diagnostic uncertainty), the educational approach and potential findings may be transferrable to other clinical settings. If findings from the study support the use of this approach, comparable curricular materials and SBML checklists could be developed to improve communication in other clinical settings. Diagnostic uncertainty is only one way in which uncertainty can shape health care decision-making. Analogous interventions may be valuable to improve communication and manage other forms of uncertainty, such as uncertainty related to treatment successes or prognoses.

Our study has several limitations. We are conducting this investigation at two residency-training sites. It is possible that the residents are not representative of a national sample. In addition, as residents in both study arms interact regularly, it is possible that there may be contamination between the control and intervention arms, which would result in an underestimation of the impact of the UCEM. The multifaceted nature of the intervention could make it difficult to discern which part of the intervention was most impactful (online modules, interactive game or SBML); however most educational interventions are multifaceted and information is often retained best when presented via multiple modalities. In addition, the use of multiple (*n* = 16) cases increases the complexity of the evaluation. While use of multiple cases was a deliberate choice of the study team to reflect the true ED environment, focusing more narrowly on one diagnosis (e.g., chest pain) and/or one emotional state (e.g., nervousness) would decrease potential unintended variation in difficulty of cases. Another potential limitation is our delivery of the deliberate practice sessions using a video tele-simulation platform. We acknowledge video-based deliberate practice has not historically been used for mastery learning, as the simulation encounters and deliberate practice are supposed to be as close as possible to real clinical experience; however, the majority of SBML has been completed for procedures where hands-on training is necessary to learn skills. Traditional in-person deliberate practice sessions were considered; but this option is not easily disseminated, and the team decided that an in-person encounter was not essential for a conversation-based skill that can be practiced via a less-resource intensive platform.

Further, as with any simulation, there is a possible lack of translation of the improved conversations in simulation into the clinical environment. The ultimate goal of this line of research is to demonstrate translation to the clinical environment; however, a necessary and foundational step before evaluating translational outcomes is to ensure that the intervention has been well designed and is effective within the simulation environment.

In conclusion, this trial will have important implications for residency training of communication skills related to diagnostic uncertainty. The trial will further have implications for design and dissemination of future SBML interventions via a telehealth platform. Further, for the nearly one in three patients who leave their ED encounter with diagnostic uncertainty, this trial will lay the groundwork for improving the quality of that clinical encounter.

## Data Availability

The intervention components of the UCEM are publically available—links provided in text. Data access will be considered upon request from Drs. McCarthy and Rising. d-mccarthy2@northwestern.edu and Kristin.rising@jefferson.edu

## Appendix

### The Uncertainty Communication Checklist

**INTRODUCTION**

1. Explain to the patient that they are being discharged.
2. Ask if there is anyone else that the patient wishes to have included in this conversation in person and/or by phone

**TEST RESULTS/ED SUMMARY**

3. Clearly state that either “**life-threatening**” or “**dangerous**” conditions have not been found.
4. Discuss diagnoses that were considered (using both medical and lay terminology).
5. Communicate relevant results of tests to patients (normal or abnormal).
6. Ask patient if there are any questions about testing and/or results.
7. Ask patient if they were expecting anything else to be done during their encounter - if yes, address reasons not done.

**“NO/UNCERTAIN DIAGNOSIS”**

8. Discuss possible alternate or working diagnoses
9. Clearly state that there is a not a confirmed explanation (diagnosis) for what the patient has been experiencing
10. Validates the patient’s symptoms
11. Discuss that the ED role is to identify conditions that require immediate attention
12. Normalize leaving the ED with uncertainty

**NEXT STEPS/FOLLOW UP**

13. Suggest realistic expectations / trajectory for symptoms
14. Discuss next tests that are needed, if any
15. Discuss who to see next AND in what timeframe

**HOME CARE**

16. Discuss a plan for managing symptoms at home
17. Discuss any medication changes.
18. Ask patient if there are any questions and/or anticipated problems related to next steps (self-care and future medical care) after discharge

**REASONS TO RETURN**

19. Discuss what symptoms should prompt immediate return to the ED

**GENERAL COMMUNICATION SKILLS**

20. Make eye contact
21. Ask patient if there are any other questions or concerns

## Abbreviations

ED: Emergency Department
MPS: Minimum Passing Standard
SBML: Simulation Based Mastery Learning
UCC: Uncertainty Communication Checklist
UCEM: Uncertainty Communication Education Module
